# Risk factors for predicting heart failure: a subject-level meta-analysis from the HOMAGE database

**DOI:** 10.1186/2049-3258-73-S1-P9

**Published:** 2015-09-17

**Authors:** Ljupcho Efremov, Lotte Jacobs, Lutgarde Thijs, Faiez Zannad, Jan A Staessen

**Affiliations:** 1 Studies Coordinating Centre, Leuven, Belgium; 2 Centre d'Investigation Clinique Pierre Drouin, Nancy, France

## Introduction

A common and potentially fatal disease, affecting notably the elderly population, is heart failure. Within the framework of the Heart OMics' in AGEing (HOMAGE) project, which aims to identify new biomarkers that can detect pathological processes that will allow early therapeutic intervention, we will conduct a subject-level meta-analysis to identify risk factors associated with new onset heart failure.

## Methods

The HOMAGE database includes 46,134 subjects from 20 studies in eight European countries and 1 American study. The database includes data from (1) prospective population studies or (2) cross-sectional, prospective studies or randomized controlled trials (RCTs) of patients at risk for or with overt cardiovascular (CV) disease. Separate analyses will be done for population studies and studies including patients at risk of CV disease.

## Results

We excluded 12 studies that do not have information on incident heart failure. From the 9 remaining studies, all patients with heart failure at baseline (n=2588) or with missing information on heart failure at baseline (n=505) were excluded. We further excluded 4 studies(STOPHF, DYDA, HVC and FLEMNGHO) because they had less than 20 incident heart failure events. In the final analysis 5 studies (n=30560) were included: 3 studies in patient at risk for CV disease(PROSPER, HULL, ASCOT) and 2 population studies in elderly (PREDICTOR, HEALTH ABC). The number of heart failure events (fatal and non-fatal combined) was 771 (3%) in the 3 patient studies and 618 (14%) in the 2 population studies. We will use Cox regression models to assess the association between candidate risk factors and incident heart failure.

## Conclusion

With our study we anticipate to identify and confirm risk factors which will help to target high-risk candidates for evaluation with the hopes of a delay in the onset or development of heart failure.

